# High resolution metagenomic characterization of complex infectomes in paediatric acute respiratory infection

**DOI:** 10.1038/s41598-020-60992-6

**Published:** 2020-03-03

**Authors:** Ci-Xiu Li, Wei Li, Jun Zhou, Bing Zhang, Yan Feng, Chang-Ping Xu, Yi-Yu Lu, Edward C. Holmes, Mang Shi

**Affiliations:** 1 Key Laboratory of Emergency Detection for Public Health of Zhejiang Province, Zhejiang Provincial Centre for Disease Control and Prevention, Hangzhou, 310021 China; 2 School of Basic Medicine, Zhejiang University, Hangzhou, 310005 China; 3 Marie Bashir Institute for Infectious Diseases and Biosecurity, School of Life and Environmental Sciences and School of Medical Sciences, The University of Sydney, New South Wales, 2006 Australia; 4 Department of Clinical Laboratory, The Children’s Hospital of Zhejiang University School of Medicine, Hangzhou, 310052 China; 5 Department of Clinical Laboratory, Hangzhou children’s hospital, Hangzhou, 310014 China; 6 College of Life Science, Zhejiang Chinese Medical University, Hangzhou, 310053 China

**Keywords:** Metagenomics, Viral infection

## Abstract

The diversity of pathogens associated with acute respiratory infection (ARI) makes diagnosis challenging. Traditional pathogen screening tests have a limited detection range and provide little additional information. We used total RNA sequencing (“meta-transcriptomics”) to reveal the full spectrum of microbes associated with paediatric ARI. Throat swabs were collected from 48 paediatric ARI patients and 7 healthy controls. Samples were subjected to meta-transcriptomics to determine the presence and abundance of viral, bacterial, and eukaryotic pathogens, and to reveal mixed infections, pathogen genotypes/subtypes, evolutionary origins, epidemiological history, and antimicrobial resistance. We identified 11 RNA viruses, 4 DNA viruses, 4 species of bacteria, and 1 fungus. While most are known to cause ARIs, others, such as echovirus 6, are rarely associated with respiratory disease. Co-infection of viruses and bacteria and of multiple viruses were commonplace (9/48), with one patient harboring 5 different pathogens, and genome sequence data revealed large intra-species diversity. Expressed resistance against eight classes of antibiotic was detected, with those for MLS, Bla, Tet, Phe at relatively high abundance. In summary, we used a simple total RNA sequencing approach to reveal the complex polymicrobial infectome in ARI. This provided comprehensive and clinically informative information relevant to understanding respiratory disease.

## Introduction

Acute respiratory infections (ARI) are a leading cause of morbidity and mortality in newborns and young children, who experience an average of 3 to 6 ARIs annually^1–3^. Identifying the diversity of pathogens responsible for ARIs remains challenging because they involve a diverse set of viruses, bacteria, and fungal pathogens, with co-infection among them commonplace^4,5^. Traditional testing methods such as PCR, serological typing, bacterial culture and antibody detection, are regarded as the “gold standard” and widely used in ARI diagnosis^6,7^. However, despite an ongoing effort to include multiple pathogens in a single assay^8,9^, it remains difficult to simultaneously identify all potential ARI pathogens and capture new or uncommon respiratory pathogens^10^.

Metagenomic next-generation sequencing (mNGS) is an unbiased way of discovering a broad range of infectious agents^11–13^, and has been recently introduced into clinical research to investigate the microbial cause of unusual disease cases^14^, perform broad-scale surveys for pathogens in undiagnosed diseases^15,16^, and understand the role of opportunistic infections^17,18^. For example, a study of severe pneumonia revealed that mNGS is both efficient and reliable^19,20^. Importantly, the utility of mNGS goes beyond pathogen identification. In particular, total RNA sequencing (“meta-transcriptomics”) has successfully revealed the entire “infectome” (viruses, bacteria and eukaryotes) present within an organism and provided relevant data on genome sequence, gene expression, and pathogen abundance^21–26^. In addition, the focus on expressed RNA means that the sequence data generated is not dominated by those from the host genome, in turn simplifying pathogen discovery.

Herein, we used meta-transcriptomics to investigate the infectious causes of ARI in 48 children attending a single hospital in China over a defined time-period. Our aim was to characterize the complex infectome of ARIs using a straightforward yet powerful approach that provides plentiful information in addition to identifying the likely pathogen, establishing a bench-mark for future studies in this area. Accordingly, the total spectrum of microbes present in patient throat swabs samples were identified, which involved both complex and diagnostically challenging cases.

## Results

### Cohort characteristics

**Figure 1:**
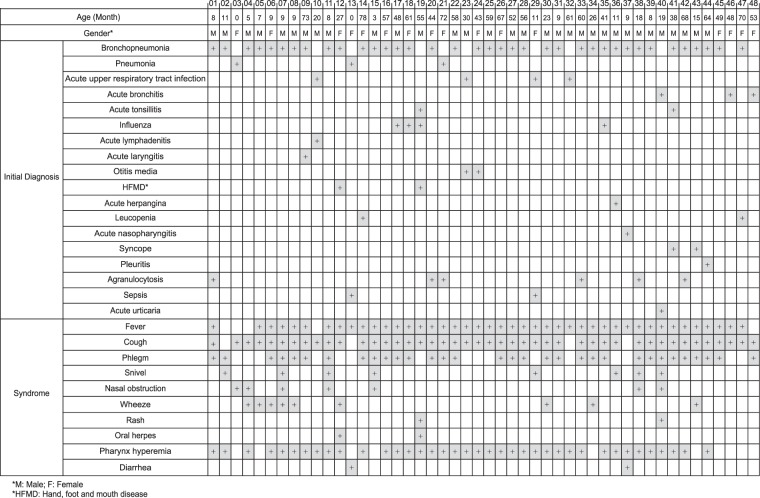
Clinical information on the paediatric ARI cohort used in this study. M = male; F = Female; HFMD = Hand, foot and mouth disease. Each positive diagnostic result or symptom is denoted with a “ + ”.

In total, throat swab samples of 48 paediatric patients and seven controls were subjected to meta-transcriptomics analysis (Fig. 1). The sex ratio of the patients was 2:1 male to female, with age ranging from neonates to 6 years (medium, 2.9 years). These patients all had final diagnoses of bronchopneumonia (37/48), pneumonia (3/48), acute upper respiratory tract infection (4/48), acute bronchitis (3/48), acute tonsillitis (2/48), or influenza (4/48) (Fig. 1). The corresponding symptoms included fever, cough, phlegm, snivel, wheeze, and nasal obstruction. Rash, oral herpes, pharynx hyperemia, and diarrhea were observed in some cases (Fig. 1).

### Characterizing total infectomes

All 55 samples (48 patients, seven controls) were examined individually using total RNA sequencing, which generated between 13.7–98.1 million reads per sample (Table S1). Downstream analyses based on reads and assembled contigs revealed a broad range of microbes, including potential pathogens likely responsible for ARI, that comprised RNA viruses, DNA viruses, bacteria, and fungi.

### Presence and abundance of viruses

**Figure 2:**
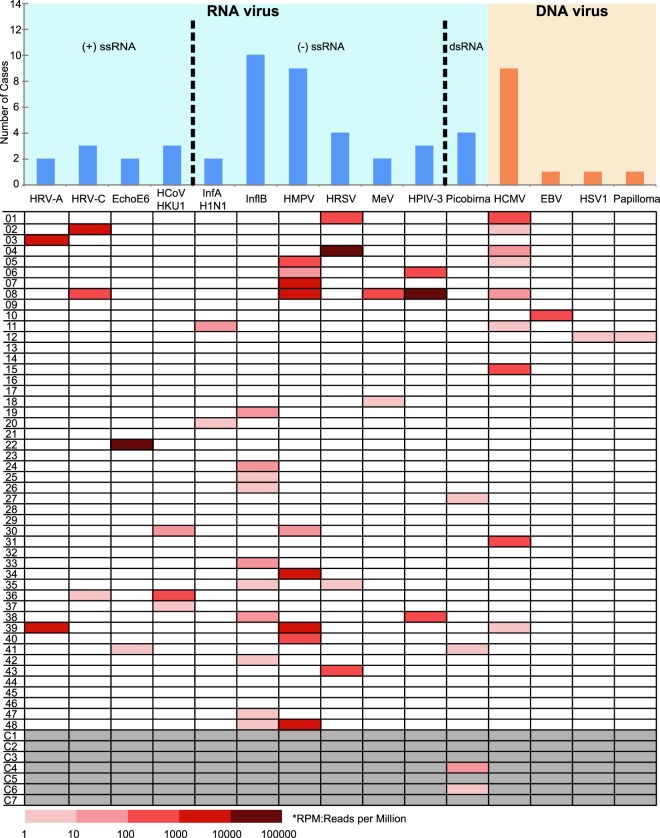
Diversity and abundance of viruses identified in this study. (**A**) Number of virus species in each case, including RNA viruses (blue) and DNA viruses (orange). (**B**) Heatmap showing the abundance level of different virus species within each library. The abundance level of reads was normalized to unique reads mapped per million input reads (RPM). HRV-A: human rhinovirus A; HRV-C: human rhinovirus C; Echo6: echovirus E6; HCoV-HKU1: human coronavirus HKU1; InfA(H1N1): influenza A virus H1N1; InfB: influenza B virus; HMPV: human metapneumovirus; HRSV: human respiratory syncytial virus; MeV: measles virus; HPIV-3: human parainfluenza virus 3; HCMV: human cytomegalovirus; EBV: Epstein-Barr virus; HSV1: herpes simples virus 1; picobirna: members of the family *Picobirnaviridae*; papilloma: members of the family *Papillomaviridae*.

Blastx comparisons against the nr database identified at least 15 virus species associated with human infection: 11 RNA viruses and four DNA viruses (Fig. 2). The total virus positive rate was 73% (35/48). Other than picobirnaviruses that are present in both case and control samples, all the other viruses are known to be disease associated, although some (such as human rhinovirus A and Epstein–Barr virus) are opportunistic pathogens frequently found in healthy populations. Among the clinically relevant viruses, influenza B virus (InfB) had the highest positivity rate (n = 10), followed by human metapneumovirus (HMPV, n = 9), human cytomegalovirus (HCMV, n = 9), human respiratory syncytial virus (HRSV, n = 4), human parainfluenza virus 3 (HPIV3, n = 3), human coronavirus HKU1 (HCoV HKU1, n = 3), rhinoviruses A (HRVA, n = 2) and C (HRVC, n = 3), and influenza A virus (H1N1, n = 2). Measles virus was detected in two of the samples, although neither of these patients exhibited symptoms compatible with measles infection, such as rash or Koplik’s spots. In addition, echovirus 6, an enterovirus not commonly with respiratory infections, was detected in two patients.

We quantified virus expression levels by estimating their relative abundance (RPM, reads per million). Accordingly, the highest abundance was 63,005 RPM (Human parainfluenza virus 3), while those lower than 1 RPM were not considered as true positives (Fig. 2). Viruses with greater than 10,000 RPM (>1% of total reads) included echovirus E6 (Case 22), HRSV (Case 4), and HPIV-3 (Case 8). Such high levels of abundance suggest active replication. In comparison, picobirnaviruses, herpes simplex virus 1, and papillomaviruses were all at very low abundance (Fig. 2). Importantly, although RNA sequencing was performed, the abundance of DNA viruses can also be quantified by estimating the abundance of the RNA transcripts they produce. Although no DNA viruses were highly abundant, in Cases 1, 10, 15, 31 the abundance levels of herpesviruses (i.e. HCMV and EBV) were greater than 100 RPM (Fig. 2).

### Virus characterization

**Figure 3:**
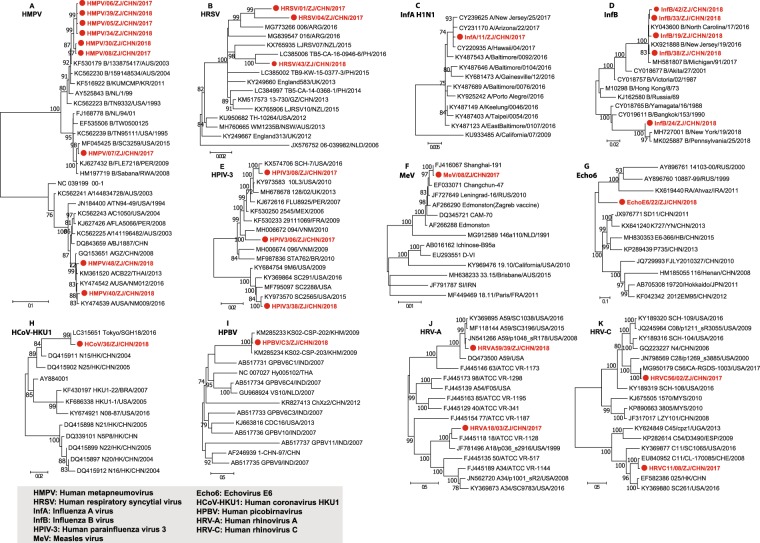
Intra-species diversity and phylogenetic relationships of RNA viruses identified in this study. Viruses identified here are marked in red and highlighted with a red solid circle, whereas those representing the background phylogenetic diversity are shown in black. All horizontal branch lengths are scaled to the number of nucleotide substitutions per site, and trees are mid-point rooted for clarity.

The meta-transcriptomic data generated here also provided information on the specific viral genotypes/lineages present in this population and their epidemiological origins (Fig. 3). Notably, multiple genotypes or subtypes were identified in a number of cases, including both the Yamagata and Victoria lineages of influenza B virus, and the A and B genotypes of HMPV (Fig. 3A,D). Conversely, phylogenetic analysis revealed distinct sequence clusters containing several very closely related genomes, indicating that these viruses might originate from the same outbreak in the Chinese population (Fig. 3A,D). To exclude contamination, all metagenomic hits to influenza B viruses and HMPV were confirmed by PCR. Interestingly, the MeV sequence in patient 8 exhibited a very close phylogenetic relationship to a Chinese vaccine strain (Shanghai-191, 99.97% nucleotide identity) (Fig. 3F): this suggests a vaccine origin, although one that has clearly replicated to high levels in this patient (702 RPM) (Fig. 2). In contrast, the echovirus 6 virus discovered here (EchoE6/22/ZJ/CHN/2018), was relatively distant to known viruses (<89.78% nt identity), such that it likely represents a new variant of this virus (Fig. 3G). The only influenza A virus identified belonged to the H1N1/09 subtype (Fig. 3C).

### Bacteria and fungi

**Figure 4:**
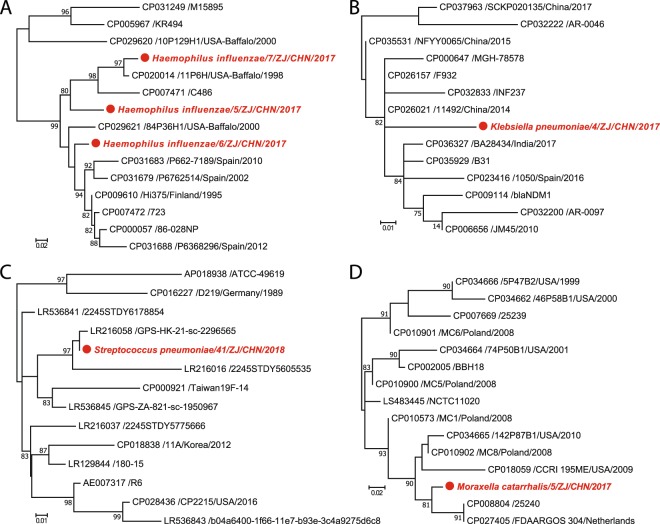
Phylogenetic analysis of *Haemophilus influenzae* (**A**), *Klebsiella pneumoniae* (**B**), *Streptococcus pneumoniae* (**C**) and *Moraxella catarrhalis* (**D**), based on the concatenated multigene data set. Within each phylogeny, the bacteria identified in this study are marked in red and highlighted with a red solid circle. All horizontal branch lengths are scaled to the number of nucleotide substitutions per site, and trees are mid-point rooted for clarity.

The dominant bacterial genera identified in these patients were *Neisseria* (20.19%, measured in RPM), *Prevotella* (19.89%), *Veillonella* (19.76%), *Leptotrichiaceae* unclassified (16.01%), *Capnocytophaga* (8.76%), *Haemophilus* (5.76%), and *Streptococcus* (2.66%) (Fig. S1). However, since relatively high bacterial abundance was observed in both patient and control samples it is difficult to distinguish commensal from potentially pathogenic bacteria at the genus level (Fig. S2). A further species level identification based on both MetaPhlAn and multiple reference gene mapping identified several disease-causing bacterial species, including *Haemophilus influenzae* (three cases), *Klebsiella pnemoniae*, *Moraxella catarrhalis*, and *Streptococcus pneumoniae* that may be responsible for some of the ARIs observed (Fig. 4 and Table 1).

**Table 1:** Identification of virus, bacteria, and fungi pathogens in ARI using meta-transcriptomics.

No.	Initial Diagnosis	Syndrome*	Initial pathogen screening (Not performed in this study)	Final diagnosis	
				Virus	Bacteria/Fungus
1	Bronchopneumonia Agranulocytosis	F; C; P; PH	*Staphylococcus aureus* (Culture) *Streptococcus viridans* (Culture)	Human respiratory syncytial virus + Human cytomegalovirus+	
2	Bronchopneumonia	P; S; PH	*Staphylococcus aureus* (Culture)	Human rhinovirus C++	
3	Pneumonia	C; NO	Human cytomegalovirus (PCR) *Staphylococcus aureus* (Culture) *Haemophilus influenza* (Culture) *Streptococcus viridans* (Culture)	Human rhinovirus A++	
4	Bronchopneumonia	C; NO; W; PH	Respiratory syncytial virus (PCR) *Klebsiella pneumoniae* (Culture)	Human respiratory syncytial virus++ + Human cytomegalovirus	*Klebsiella pnemoniae* +
5	Bronchopneumonia	F; C; W		Human metapneumovirus+	*Haemophilus influenzae* *Moraxella catarrhalis*
6	Bronchopneumonia	F; C; P; W; PH		Human metapneumovirus Human parainfluenza virus 3+	*Haemophilusm influenzae*++
7	Bronchopneumonia	F; C; P; S; NO; W; PH		Human metapneumovirus++	*Haemophilus influenzae*+
8	Bronchopneumonia	F; C; P; W; PH	*Staphylococcus aureus* (Culture) *Streptococcus viridans* (Culture) *Haemophilus parainfluenzae* (Culture)	Human rhinovirus C + Human metapneumovirus + + Measles virus + Human parainfluenza virus 3 + + + Human cytomegalovirus	
10	Acute upper respiratory tract infection Acute lymphadenitis	C; PH	Epstein–Barr virus (PCR, Antibody)	Epstein–Barr virus+	
11	Bronchopneumonia	F; C; P; S; NO; PH	*Haemophilus influenza* (Culture)	Influenza A (H1N1)	
12	Bronchopneumonia HFMD	F; C; W; OH; PH	Yeast (Culture)		*Candida sp*.
13	Pneumonia Sepsis	F; D	Gram positive bacteria		
15	Bronchopneumonia	F; C; P; S; NO	Human cytomegalovirus (Antibody) *Staphylococcus aureus* (Culture) *Neisseria sp*. (Culture) *Streptococcus viridans* (Culture)	Human cytomegalovirus+	
18	Bronchopneumonia Influenza	F; C; P; PH	Influenza B virus (PCR)		
19	Acute tonsillitis Influenza	F; C; R; OH; PH	Enterovirus (universal) positive (PCR)	Influenza B virus	
20	Bronchopneumonia Agranulocytosis	F; C; P; PH	*Staphylococcus aureus* (Culture) *Streptococcus viridans* (Culture)		
22	Bronchopneumonia	F; C; P; PH		Echovirus E6+++	
23	Acute upper respiratory tract infection Otitis media	F; C; PH		Influenza B virus	
30	Bronchopneumonia	F; C; P; W; PH		Human coronavirus HKU1 Human metapneumovirus	
31	Bronchopneumonia	F; C; P; PH		Human cytomegalovirus+	
33	Bronchopneumonia Agranulocytosis	F; C; P; PH		Influenza B virus	
34	Bronchopneumonia	F; C; P; W		Human metapneumovirus++	
36	Bronchopneumonia Acute herpangina	F; C; S; PH		Human coronavirus HKU1+	
38	Bronchopneumonia Agranulocytosis	F; C; P; S; NO; PH		Influenza B virus Human parainfluenza virus 3+	
39	Bronchopneumonia	F; C; P; PH		Human rhinovirus A+ + Human metapneumovirus + +	*Streptococcus pneumoniae*
40	Acute tonsillitis Acute urticaria	F; C; P; S; NO; R; PH	*Staphylococcus aureus* (Culture) *Gram positive bacteria* (Culture) *Streptococcus viridans* (Culture)	Human metapneumovirus +	
43	Bronchopneumonia Syncope	F; C; P; W		Human respiratory syncytial virus +	
48	Acute bronchitis	C; P		Human metapneumovirus + +	

*F, Fever; C, Cough; P, Phlegm; S, Snivel; NO, Nasal obstruction; W, Wheeze; R, Rash; OH, Oral herpes; PH, Pharynx hyperemia; D, Diarrhea.

**Figure 5:**
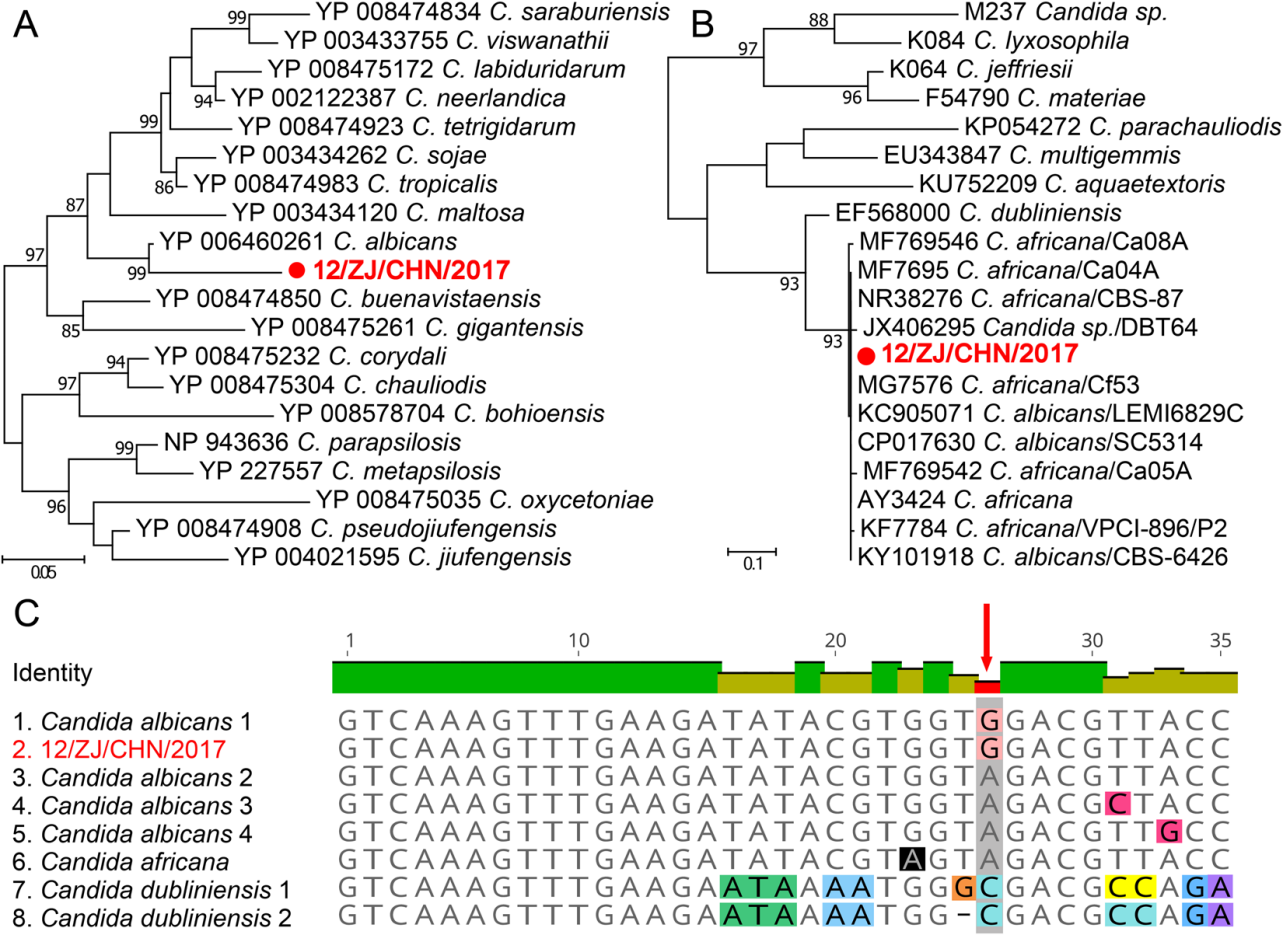
Identification of a novel fungal species *Candida* sp. Phylogenetic trees are based on the cytochrome c oxidase subunit 1 (COX 1) gene (**A**) and the internal transcribed spacer region (ITS) (**B**). Species identified in this study are marked in red. (**C**) Signature sequences of a short fragment (35 bp) of the internal transcribed spacer region 2 (ITS2). Sequences are aligned against the 35 bp signature of the most common *C. albicans* variant (*C. albicans* 1) (34). Coloured letters indicate positions in which the sequence differs from that of *C. albicans* 1. The red arrow indicates position that species identified in this study differs from that of *C. albicans* 1.

Notably, a fungal infection was identified in one patient (Case 12), for which the initial culturing of 10 samples identified a yeast-like organism (Table 1). This was validated in our metagenomic study, with the sequencing reads mapping to fungi of the genus *Candida*. Subsequently, we retrieved the cytochrome c oxidase subunit 1 (COX 1) gene and an internal transcribed spacer region (ITS, 375 bp)^27^ from the assembled contigs of the fungus species and utilized these in phylogenetic analyses: this revealed that the newly identified fungus was closely related to *C. Africana* and C. *albicans* (Fig. 5). However, its position in the COX1 phylogeny and its divergence (93.33%) suggests that it represents a new fungal species.

### Microbial co-infections

In total, 28 of the 48 cases harboured at least one potentially pathogenic microbe that are likely responsible for the ARIs observed (Table 1). Notably, among these, nine cases were co-infected with more than two pathogens, five had virus and bacteria co-infections, seven had multiple virus infections, and in one case there was evidence for the presence of at least five different viral species. Microbial co-infections were therefore commonplace in these patients. The virus and bacteria species involved in co-infections were diverse, although it is notable that HMPV and *Haemophilus influenzae* co-appeared in three cases (Table 1).

### Comparison with other diagnostic methods

As well as our meta-transcriptomic analysis, all the patients studied here were subjected to other pathogen screening protocols performed at the Hangzhou Children’s Hospital. These diagnostic tests included bacterial/fungal culture, PCR, real-time PCR, and antibody-based testing, using either the same (i.e. oral swaps) or different (e.g. sputum, blood) samples as the metagenomics. Notably, a large proportion of the viruses, especially RNA viruses, were not identified in the diagnostic laboratory tests, and sometimes the tests were unavailable or not performed. Consequently, many cases with high viral loads (e.g. Cases 1–4, 8, and 30) were diagnosed as bacterial infections (Table 1). In the case of bacteria and fungi the meta-transcriptomics and standard diagnostic tests returned both consistent (Cases 4 and 12) and inconsistent (Cases 1–3, 5–8, 11, 15, 20, and 40) results, although meta-transcriptomics had a lower bacterial detection rate (Table 1).

### Diversity and abundance of antibiotic resistance genes (ARGs)

**Figure 6:**
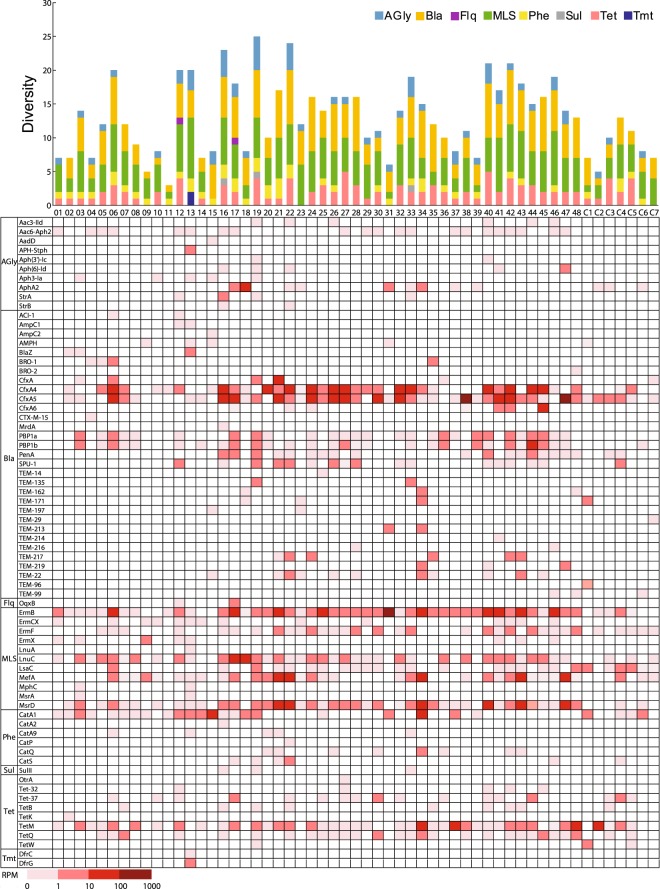
Antibiotic resistance genes expressed in the samples collected from ARI patients and control cases. (**A**) Diversity of resistance genes observed in each library. Tmt = Trimethoprim, Tet = Tetracyclines, Sul = Sulfonamides, Phe = Phenicols, MLS = Macrolide-Lincosamide-Streptogramin, Flq = Fluoroquinolones, Bla = β- lactamases, and AGly = Aminoglycosides. (**B**) The abundance level of each antibiotic resistance gene identified in this study. These genes are grouped into 8 classes based on their resistance target and their abundance levels were measured using RPM.

We used the meta-transcriptomic data generated here to screen for the presence and relative abundance of ARGs. After removing those ARGs likely associated with cloning vectors (e.g. TEM-116 and TetC), our analyses revealed the expression of resistance genes against eight classes of antibiotics: aminoglycosides (AGly), beta-lactamases (Bla), fluoroquinolones (Flq), macrolide-lincosamide-streptogramin (MLS), phenicols (Phe), sulfonamides (Sul), tetracyclines (Tet), trimethoprim (Tmt) (Fig. 6). Strikingly, there was a significant difference (P < 0.05, Fig. S3) in the diversity and abundance of ARGs between cases and controls. The number of ARGs in each case varied from 1–25, with the total abundance level 0.11–263.16 RPM, whereas for the controls the numbers varied from 5–13, with abundance ranging from 0.11–13.78 (Fig. 6).

### Individual cases

We now discuss a number of the individual cases in more detail, each highlighting different aspects of the complex microbial basis of ARI.

#### Multiple virus infections

Case 8 was a 9-month boy who presented with persistent cough of seven days duration followed by fever. He was twice admitted to hospital with bronchitis before sampling, and was diagnosed with bronchopneumonia on his second visit. A blood examination revealed a WBC count of 18.89/ul, including 33.1% neutrophils, 55.8% lymphocytes, a HGB 121 g/l, Plt 332ul and CRP 85 mg/L. The initial laboratory test suggested that *Staphylococcus aureus*, *Viridans streptococci* and *Haemophilus influenzae* were present in this patient, none of which were confirmed with our meta-transcriptomics analysis. Instead, five different viruses were detected in this patient, including three that are likely pathogenic (HRV-C, HPIV3, and HMPV), one vaccine related (measles virus), as well as one opportunistic virus (HCMV). These viruses all had medium to high abundance (48.07–63005.37 RPM) (Figs. 2 and S4), indicative of active replication. HPIV3 had the highest abundance and is commonly associated with bronchiolitis and pneumonia. The disease manifestation was not severe, no measles specific syndromes were recorded, and the patient was discharged after five days.

#### Fungal infection

Case 12 was 2-year-old girl with seven days of fever (39 °C) and one day of cough who presented with a typical hand, foot and mouth disease (HFMD) syndrome. However, a PCR and antibody test against EV71, Coxsackievirus A16 and enteroviruses (universal) were negative. A throat culture followed by microscopy suggested the presence of a yeast-like agent, which was identified as a member of the genus *Candida* by phylogenetic analyses (see above; Fig. 5). The species identified is closely related to *C. albicans* and *C. Africana*, often associated with a condition called “thrush”.

#### Enterovirus infection

Case 20 was 5-year-old boy who experienced seven days paroxysmal cough and two of days fever, as well as paroxysmal attacks of frontal pain. Blood and throat swab cultures for bacterial and fungal pathogens were all negative, as were PCR and antibody tests for common respiratory viruses. Strikingly, our meta-transcriptomic analyses identified a divergent variant of echovirus 6 present at high abundance (11382.76 RPM) (Fig. 2). Echovirus 6 is a member of enterovirus group B^28^, and associated with aseptic meningitis, herpangina, HFMD, and sometimes respiratory disease: hence, this is the likely pathogen in this particular case.

#### Coronavirus infection

Case 36 was an 11-month-old boy who presented with seven days of fever (>38 °C) and cough. PCR and antibody tests against common respiratory viruses were all negative, and a throat swab culture found no evidence of bacterial or fungal pathogens. In contrast, meta-transcriptomics identified human coronavirus HKU1, which is the likely pathogen in this case (Fig. 2 and Table 1). Importantly, this virus is often excluded from the PCR panel used for respiratory disease, thereby illustrating the utility of meta-transcriptomics.

## Discussion

We used an expansive meta-transcriptomics approach to characterize the microbial pathogens associated with paediatric ARI. By revealing a diverse array of pathogens that are often not included in standard laboratory diagnostics we demonstrated that total RNA sequencing is an efficient, accurate and powerful means to characterize the infectome of a target tissue in clinical cases.

Importantly, since NGS-based pathogen discovery involves complicated multistep protocols, contamination can be introduced from a variety of sources such as the laboratory environment, the reagents used, and from multiplexing samples in a single sequencing run^29,30^. To reduce the likelihood of contamination in this study, we utilised (i) case controls, (ii) detailed genetic analyses involving full genome or multiple genes, (iii) PCR confirmation in the case of closely related sequences, and (iv) additional evidence whenever a new species was identified, such as the use of microscopy in the case of the novel fungal pathogen.

Not only did our meta-transcriptomic analysis reveal a diversity of RNA viruses, but also DNA viruses, bacteria and fungi through their expressed RNA. In addition to those pathogens routinely screened, we were able to identify a novel fungal pathogen, a divergent variant of an atypical respiratory virus (echovirus 6), a replicating vaccine strain of measles virus, and an array of pathogens not commonly included in the PCR panels routinely used for respiratory pathogens, such as coronaviruses, rhinoviruses, and herpesviruses. Meta-transcriptomics also provided substantial additional biological information, including pathogen abundance (a marker of replication activity and infectivity), genome sequence data that informs on epidemiological and evolutionary origins, and information on antimicrobial resistance genes that will assist treatment plans.

Also striking was that ARI was often associated with multiple viruses or the co-occurrence of both viral and bacterial pathogens within a single patient. Synergistic interactions have been frequently described between bacteria and viruses, although their importance to disease manifestation requires further examination^5,31^. As a case in point, the patients studied here that harboured more than two likely pathogens did not experience an elevated disease severity.

A notable result was the marked difference between the pathogens detected by meta-transcriptomics and those determined by more routine laboratory diagnostics. As might be expected given the focus on RNA^26^, meta-transcriptomics generated a much larger diversity of viruses, especially those with RNA genomes. In addition, the assays for RNA viruses are costly and time-consuming for most hospital laboratories, and there are insufficient diagnostical tests to cover the entire diversity of respiratory viruses. Conversely, more bacteria were discovered using traditional culture methods, which is also likely true of shotgun DNA sequencing-based metagenomics^32^. This suggests that RNA-based meta-transcriptomics can be subject to false-negative results in some instances. This likely occurs because metagenomic bacterial identification was based on non-rRNA genes whose RNA expression level is often relatively low^25^. However, because they may be indicative of more elevated replication, those bacteria identified by meta-transcriptomics are perhaps more likely relevant to disease manifestation.

In sum, our study described a straightforward and powerful way to investigate the full infectome of paediatric ARIs, establishing an bench-mark for this important disease syndrome. Despite this, it is clear that studies based on larger sample sizes are necessarily required for a more complete understanding of the microbial basis of ARIs in different patient populations.

## Methods

### Ethics statement

This project was approved by the ethics committee of the Zhejiang Provincial Centre for Disease Control and Prevention, China. The need for informed consent from parents/guardians was waived by the ethics committee of the Zhejiang Provincial Centre for Disease Control and Prevention because the analyses were performed retrospectively following standard diagnostic tests, posing no extra patient burden. In addition, all data were de-identified and anonymous. All human-related sample processing and sequencing were performed in accordance with relevant guidelines and regulations of the Zhejiang Provincial CDC.

### Respiratory sample collection

We studied children with ARIs admitted to Hangzhou Children’s Hospital (Zhejiang, China) between March 2017 and January 2018. The inclusion criteria were inpatient age between 0 and 10 years who presented with at least two of the following: cough, fever, snivel, sneeze, pharyngeal discomfort, and nasal obstruction, and who were diagnosed with either bronchopneumonia, pneumonia, acute bronchitis, or acute upper respiratory tract infection. Throat swabs were collected as part of the diagnostic routine protocol, and among these 48 samples were selected for meta-transcriptomics. For comparison, samples of 7 healthy subjects were collected between October 2017 - February 2018 and utilized as controls. All specimens were collected by sterile flocked swab and were immediately sent to the hospital laboratory where they were stored at −20 °C for less than 24 hours. They were later transferred on dry ice to a −80 °C freezer.

### RNA extraction, library construction and sequencing

Samples were subjected to RNA extraction using TRlzol LS reagent (Invitrogen, USA). After obtaining total RNA, purification steps were performed using the RNeasy Plus Mini Kit (Qiagen, USA) according to the manufacturer’s instructions. The concentration and quality of final extractions were examined using a NanoVue plus spectrophotometer (GE Healthcare, UK). In all cases library preparation kits that target low-concentration RNA samples (as low as 500 pg), including the SMARTer® Stranded Total RNA-Seq Kit v2 - Pico Input Mammalian (Takara Bio, USA) and the Trio RNA-Seq kit (NuGEN Technologies, USA) were used. Paired-end (150 bp) sequencing of these RNA library was performed using the Illumina HiSeq platform, generating between 4.12–29.44 Gbp data for each library/case (Table S1).

### Pathogen characterization

For each library/case, we performed quality control and removed adaptor sequences and low-quality/low-complex reads. Human reads were removed by mapping to the human genome. All non-human sequence reads generated here have been deposited on the NCBI Sequence Read Achieve (SRA; BioProject accession PRJNA540900). These reads were then compared to the non-redundant protein database using Diamond^33^, as well as to a reference virus database downloaded from GenBank using blastn. Blast hits were sorted taxonomically to the species level by matching the accession number with the taxonomy database. Virus reads were then *de novo* assembled using Megahit^34,35^, with virus identified based on the blast procedure described above. In cases with low genome coverage, reads were directly mapped to the sequence of a close relative, and a consensus genome was obtained from the mapped reads. The resulting complete or partial virus genomes (Table S2) were aligned with related viruses from GenBank using MAFFT version 7^36^, and subjected to phylogenetic analysis using the maximum likelihood method in PhyML 3.0^37^, employing the General Time Reversible (GTR) model of nucleotide substitution with a gamma distribution of among-site rate variation, and 1000 bootstrap replicates.

To estimate virus abundance, reads were mapped back to each virus genome, and the relation “mapped reads/total reads * one million” was used to calculate the number of Reads Per Million (RPM). To exclude contamination due to index hopping, for each virus only those present at >0.1% of the highest viral abundance were considered true positives. For viruses in which highly similar sequences appeared in multiple libraries (i.e. influenza B virus, human metapneumovirus), RT-PCR and Sanger sequencing were performed to confirm their presence.

Bacterial and fungal pathogens were initially identified using MetaPhlAn2^38^, which mapped the non-human sequence reads to a set of ∼1 million marker genes from >7500 microbial species. These results were confirmed using phylogenetic analyses based on multiple gene sets (bacteria, with reference sequences downloaded from https://pubmlst.org/; Table S3) and the COX1 gene (fungi). All sequences were obtained from either the assembled contigs (blastx) or by direct mapping to a reference gene set. All genes associated with antibiotic resistance (ARGs) were first identified by Short Read Sequencing Typing (SRST2)^39^. To confirm that ARGs did not originate from cloning vectors, the assembled contigs were blasted against the nt database. The diversity and abundance of ARGs among the cases and controls were compared using a t-test.

## Supplementary information

Supplementary information.

Supplementary dataset.

## Data Availability

All non-human sequence reads generated have been deposited on the NCBI Sequence Read Achieve (SRA) database (BioProject accession PRJNA540900). And all sequence alignments used for phylogenetic analyses have been included as Supplementary Data.
