# Supplementary material for: High resolution metagenomic characterization of complex infectomes in paediatric acute respiratory infection
Source: Sci Rep. 2020 Mar 3;10:3963. doi: 10.1038/s41598-020-60992-6 (PMC7054269; doi:10.1038/s41598-020-60992-6)
Supplement: Supplementary file 1 — Supplementary information. [file 41598_2020_60992_MOESM1_ESM.pdf]

Supplementary Material

High resolution metagenomic characterization of complex infectomes in paediatric acute respiratory infection

Ci-Xiu Li[a,b,c], Wei Li[d], Jun Zhou[e], Bing Zhang[f], Yan Feng[a], Chang-Ping Xu[a], Yi-Yu Lu[a], Edward C. Holmes[c#], Mang Shi[c#]

[a]Key Laboratory of Emergency Detection for Public Health of Zhejiang Province, Zhejiang Provincial Centre for Disease Control and Prevention, Hangzhou 310021, China.
[b]School of Basic Medicine, Zhejiang University, Hangzhou 310005, China.
[c]Marie Bashir Institute for Infectious Diseases and Biosecurity, School of Life and Environmental Sciences and School of Medical Sciences, The University of Sydney, New South Wales 2006, Australia.
[d]Department of Clinical Laboratory, The Children's Hospital of Zhejiang University School of Medicine, Hangzhou 310052, China.
[e]Department of Clinical Laboratory, Hangzhou children's hospital, Hangzhou 310014, China.
[f]College of Life Science, Zhejiang Chinese Medical University, Hangzhou 310053, China.

[#]Correspondence to:

Dr. Mang Shi
Marie Bashir Institute for Infectious Diseases & Biosecurity,
Charles Perkins Centre,
School of Life & Environmental Sciences,
The University of Sydney | Sydney | NSW | 2006 | Australia
T +61 406039495
E mang.shi@sydney.edu.au

Prof. Edward C. Holmes
Marie Bashir Institute for Infectious Diseases & Biosecurity,
Charles Perkins Centre,
School of Life & Environmental Sciences,
The University of Sydney | Sydney | NSW | 2006 | Australia
T +61 2 9351 5591
E edward.holmes@sydney.edu.au

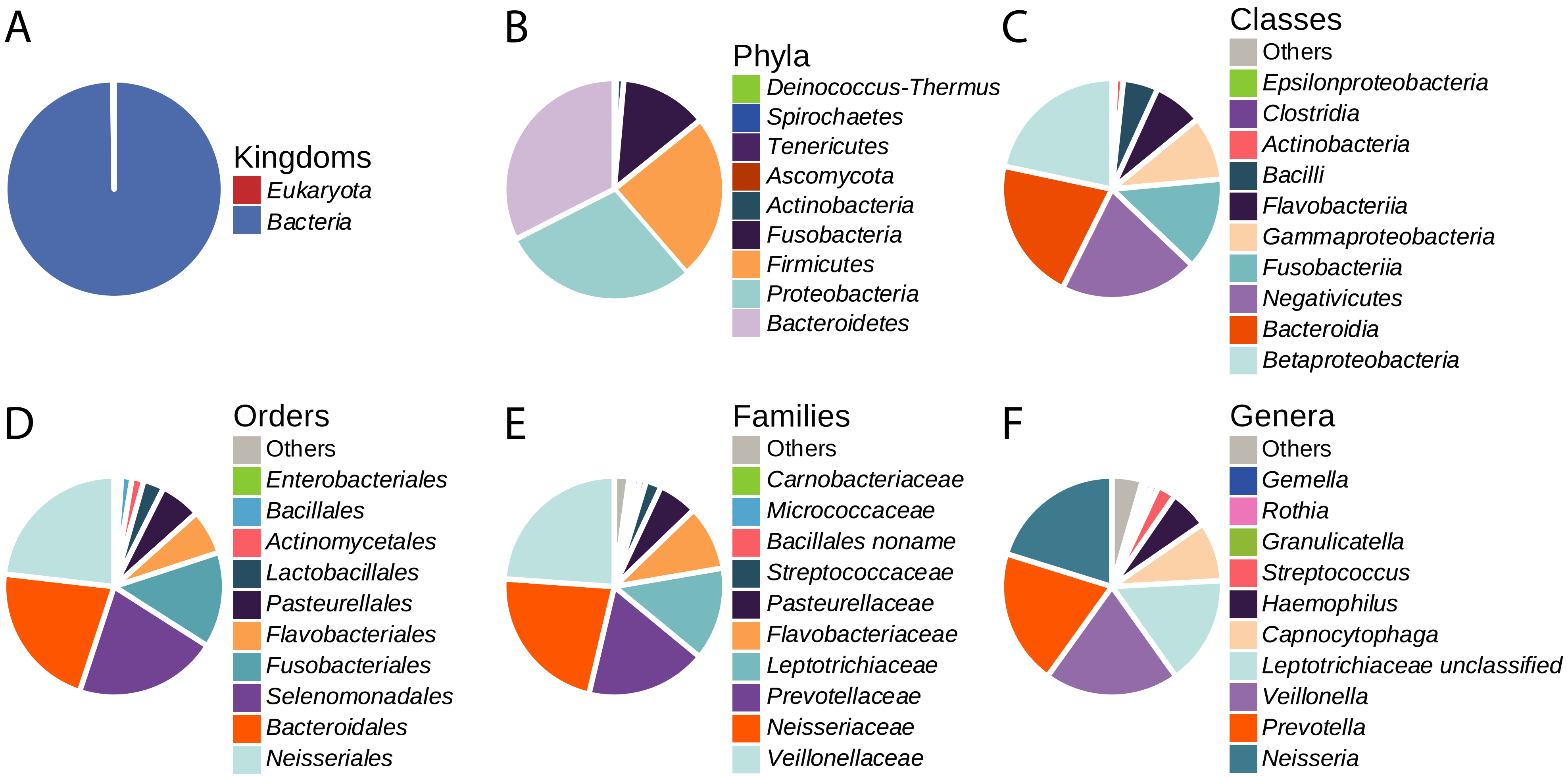

Supplementary figure S1. Profiling the relative abundance of the bacteria identified here at the kingdom (A), phylum (B), order (C), class (D), family (E) and genus levels (F). Abundance levels were estimated using MetaPhlAn2. Only the top 10 taxonomic units are shown in the pie chart.

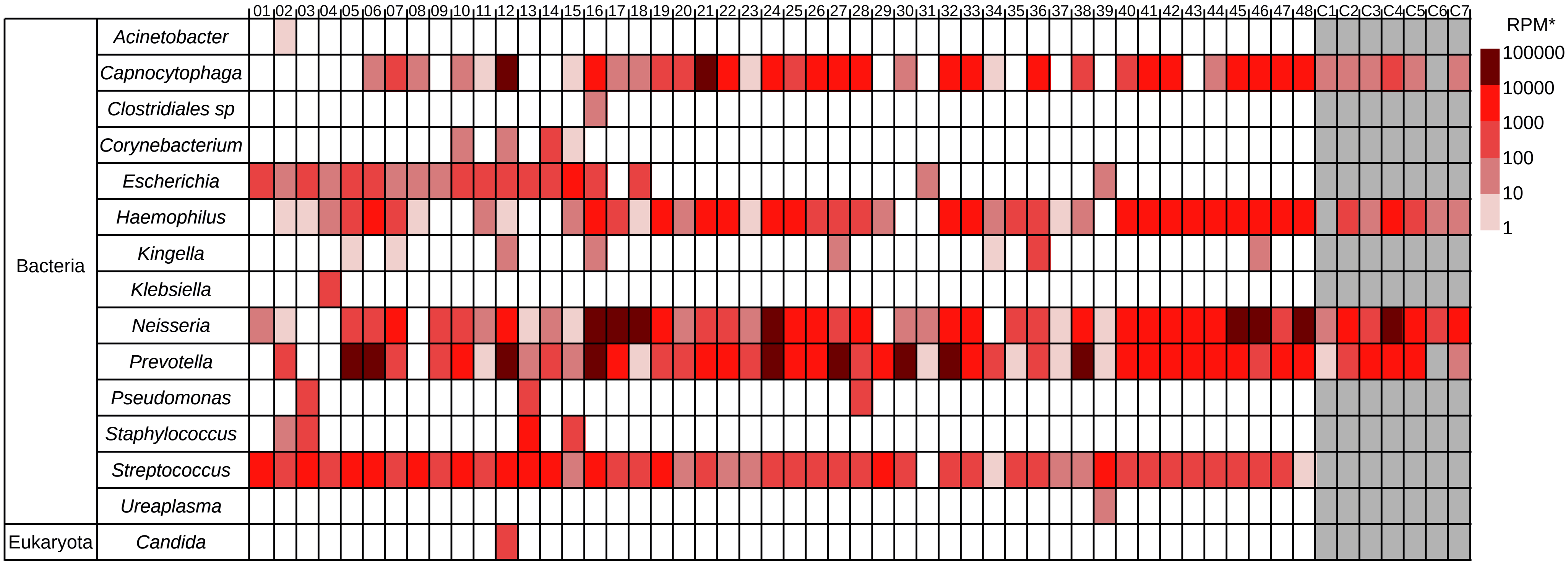

Supplementary figure S2. Heatmap showing the abundance level for the bacteria and eukaryotes identified in each case studied here. The abundance level of microbial reads was normalized to RPM, and the estimation was performed at the genus level. A total of 14 genera of bacteria and one genus of fungi were described here.

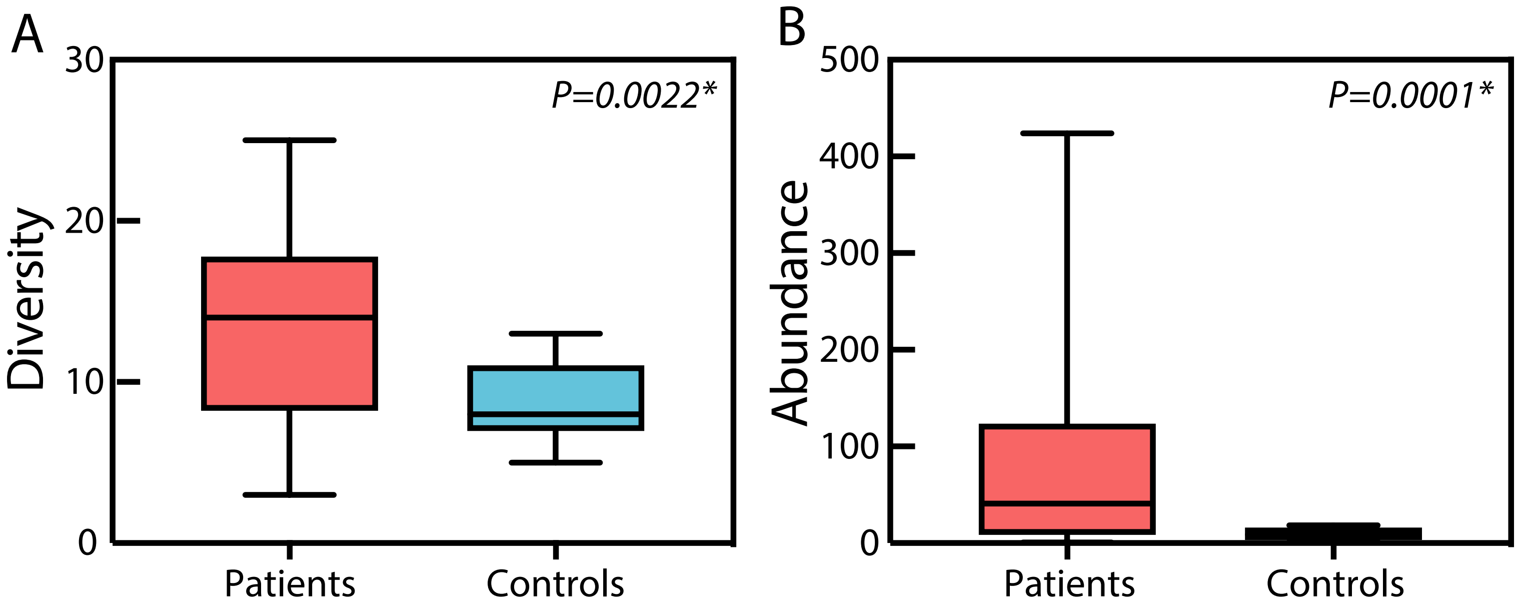

Supplementary figure S3. Comparisons of the diversity and abundance of antibiotic resistance genes (ARGs) between case and control patients. The comparisons were based on the (A) number of antibiotic resistance genes, and (B) the abundance of resistance genes. Differences between groups were assessed with a t-test.

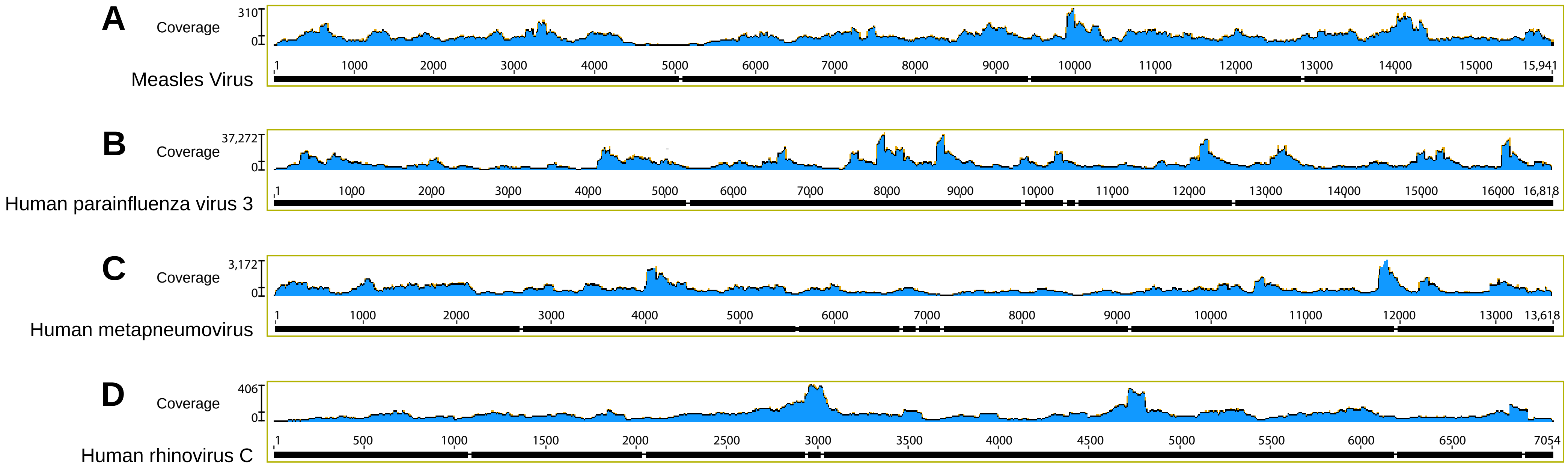

Supplementary figure S4. Mapped reads to the genomes of measles virus (A), human parainfluenza virus 3 (B), human metapneumovirus (C) and human rhinovirus C (D). A coverage range was shown to the left of each mapping results.

Supplementary table S1. Summary details of each RNA sequencing library.

Library	Total reads	Total human reads	Total non-human reads	Read Length	Total bp
1	13720028	12893390	826638	PE150	4116008400
2	44965534	44170072	795462	PE150	13489660200
3	63260278	53776732	9483546	PE150	18978083400
4	30224360	28333420	1890940	PE150	9067308000
5	43381008	36383305	6997703	PE150	13014302400
6	40125070	24497662	15627408	PE150	12037521000
7	31066310	26811133	4255177	PE150	9319893000
8	17870936	15794838	2076098	PE150	5361280800
9	22572904	21411541	1161363	PE150	6771871200
10	64325810	60069798	4256012	PE150	19297743000
11	49740216	48624878	1115338	PE150	14922064800
12	42829038	25664240	17164798	PE150	12848711400
13	47774040	43323126	4450914	PE150	14332212000
14	53820772	50558312	3262460	PE150	16146231600
15	59202148	56100033	3102115	PE150	17760644400
16	46447012	17870362	28576650	PE150	13934103600
17	48631320	562943	48068377	PE150	14589396000
18	64680372	3847632	60832740	PE150	19404111600
19	52473228	3262411	49210817	PE150	15741968400
20	43894800	2742853	41151947	PE150	13168440000
21	50961642	789286	50172356	PE150	15288492600
22	54326366	3328238	50998128	PE150	16297909800
23	50391736	37338877	13052859	PE150	15117520800
24	43437228	4545972	38891256	PE150	13031168400
25	48069620	421055	47648565	PE150	14420886000
26	43526606	415581	43111025	PE150	13057981800
27	51400146	288406	51111740	PE150	15420043800
28	35711310	20343781	15367529	PE150	10713393000
29	39083638	801190	38282448	PE150	11725091400
30	59910988	4887011	23921693	PE150	17973296400
31	45904228	17170107	28734121	PE150	13771268400
32	48263966	318900	47945066	PE150	14479189800
33	45852554	834897	45017657	PE150	13755766200
34	48578218	14184511	34393707	PE150	14573465400
35	43963124	30708813	13254311	PE150	13188937200
36	39512976	9302331	30210645	PE150	11853892800
37	57178342	48381495	8796847	PE150	17153502600
38	45131842	630942	44500900	PE150	13539552600
39	46369350	22764440	23604910	PE150	13910805000
40	44574204	640509	43933695	PE150	13372261200
41	46820986	1001851	45819135	PE150	14046295800
42	50718000	133469	50584531	PE150	15215400000
43	54234298	336370	53897928	PE150	16270289400
44	47262390	1961532	45300858	PE150	14178717000
45	40936702	172600	40764102	PE150	12281010600

46	54253766	381158	53872608	PE150	16276129800
47	48091182	403978	47687204	PE150	14427354600
48	45086692	8365564	36721128	PE150	13526007600
C1	86140276	54047353	32092923	PE150	25842082800
C2	81073142	65626043	15447099	PE150	24321942600
C3	98144526	26599147	71545379	PE150	29443357800
C4	70118314	7627912	62490402	PE150	21035494200
C5	66374814	16113213	50261601	PE150	19912444200
C6	96704894	51135668	45569226	PE150	29011468200
C7	94472054	82503310	11968744	PE150	28341616200

Supplementary table S2. Genes or genome alignments used in the phylogenetic analysis.

Virus name	Domain	Alignment length (bp)
Human metapneumovirus	Fusion glycoprotein	1620
Human respiratory syncytial virus	Near complete genome	14968
Influenza A virus	Hemagglutinin	1698
Influenza B virus	Hemagglutinin	1789
Human parainfluenza virus 3	Near complete genome	15294
Measles virus	Near complete genome	15859
Echovirus E6	Near complete genome	7352
Human coronavirus HKU1	Spike protein	4056
Human picobirnavirus	Segment 2 (replicase)	1526
Human rhinovirus A	Near complete genome	6449
Human rhinovirus C	Near complete genome	6446

Supplementary table S3. Information on the multiple gene sets used in the phylogenetic analysis of bacterial species.

Haemophilus influenzae		
	Gene	Length(bp)
	adk	443
	atpG	417
	frdB	468
	mdh	372
	pgi	320
	recA	378
	Total	2400
Klebsiella pneumoniae		
	gapA	408
	infB	293
	mdh	389
	gpi	412
	rpoB	488
	tonB	123
	Total	2131
Moraxella catarrhalis	ppa	354
	adk	287
	efp	284
	abcZ	234
	trpE	212
	fumC	145
	Total	1700
Streptococcus pneumoniae	glck	466
	spsB	458
	zwf	451
	xpt	309
	ddL	307
	aroE	155
	tkt2	139
	Total	2286
